# Low-dose extracorporeal shock wave attenuates sepsis-related acute lung injury by targeting mitochondrial dysfunction and pyroptosis crosstalk in type II alveolar epithelial cells

**DOI:** 10.3389/fimmu.2025.1637378

**Published:** 2025-08-21

**Authors:** Jianning Guo, Xiaoxuan Qu, Ruidong Ge, Die Liu, Jing Liu, Qin Hui, Fang Ye, Yuanmei Chen, Chao Wang, Di Lv, Lijuan Tang, Meihong Xia, Qi Zhang

**Affiliations:** ^1^ Department of Pediatrics, China-Japan Friendship Hospital, Beijing, China; ^2^ Department of Rehabilitation Medicine, China-Japan Friendship Hospital, Beijing, China; ^3^ China-Japan Friendship Hospital (Institute of Clinical Medical Sciences), Chinese Academy of Medical Sciences & Peking Union Medical College, Beijing, China; ^4^ Institute of Clinical Medical Sciences, China-Japan Friendship Hospital, Beijing, China

**Keywords:** shock wave, sepsis, acute lung injury, type II alveolar epithelial cell, mitochondrial DNA, pyroptosis

## Abstract

**Introduction:**

The pathological mechanism of sepsis-related acute lung injury (ALI) is closely linked to mitochondrial dysfunction and pyroptosis. Although low-dose extracorporeal shock wave (SW) therapy has been widely utilized in tissue and organ injury repair, its role in sepsis-related ALI remains unclear. This study aimed to elucidate the regulatory mechanisms of SW on mitochondrial pyroptosis crosstalk in septic ALI.

**Methods:**

The sepsis-related ALI mouse model was induced by tail vein injection of LPS. *In vitro*, LPS and ATP induced a pyroptosis model in type II alveolar epithelial (AT2) cells. The levels of inflammatory factors and oxidative stress were detected. The ultrastructure of lung mitochondria was observed by transmission electron microscope. Moreover, the mitochondrial membrane potential, ATP content, and the level of mtDNA were determined in cells and tissues. Western blot was used to detect mitochondrial oxidative stress and dysfunction, as well as the expression of pyroptosis-related proteins mediated by NLRP3 inflammasome.

**Results:**

SW significantly reduced the secretion levels of inflammatory factors TNF-α, IL-1β, IL-6, and IL-8 in serum, alveolar lavage fluid (BALF), and cell supernatant, inhibited oxidative stress markers (ROS, MDA, MPO), and upregulated antioxidant index (SOD, GSH). Pathological evidence indicates that SW can alleviate the pathological changes of lung injury and restore the mitochondrial ultrastructure of AT2 cells. The mechanism study shows that SW can enhance mitochondrial membrane potential and ATP production, inhibit mtDNA migration and p65 nuclear translocation, and down-regulate the expression of mitochondrial coding genes (MT-ND2, MT-ND4) and iNOS. At the same time, SW inhibited the NLRP3/ASC/Caspase-1 signaling axis, thereby disrupting pyroptosis cascades.

**Conclusion:**

This study reveals that SW attenuates septic ALI by targeting mitochondrial-pyroptosis crosstalk, offering a novel non-invasive therapeutic strategy for clinical applications.

## 1 Introduction

Sepsis is a life-threatening pathological syndrome characterized by organ dysfunction caused by a dysregulated host response to infection. It manifests as persistent hyperinflammation in the early phase and immunosuppression in later stages. During sepsis-induced multi-organ dysfunction, the lung is the earliest and most vulnerable target organ, with patients highly susceptible to developing acute lung injury (ALI), which is also the most common cause of sepsis-related mortality (1).Systemic inflammatory responses and oxidative stress in sepsis trigger a “cytokine storm,” amplifying inflammatory cascades that damage alveolar epithelial cells and the alveolar-capillary barrier, impairing gas exchange and exacerbating lung injury (2). In sepsis patients, mitochondrial dysfunction, outer membrane rupture, and decreased respiratory chain enzyme activity lead to cellular energy exhaustion and a disorder in the tissue cell repair mechanism. Consequently, mitochondrial DNA (mtDNA) is released into the cytoplasm. Clinical studies have demonstrated significantly elevated plasma mtDNA levels in sepsis patients, which correlate positively with the severity of organ injury (3, 4). As a damage-associated molecular pattern (DAMP), mtDNA exacerbates inflammatory responses in sepsis by activating the NLRP3 inflammasome and triggering pyroptosis, causing the death of alveolar macrophages and alveolar endothelial cells (5, 6). Although pyroptosis is another form of programmed necrosis, its abnormal regulation further exacerbates inflammatory damage, forming a vicious circle (7–9). Therefore, targeting the mitochondrial pyroptosis axis may represent a critical therapeutic strategy for sepsis-related ALI.

Low-energy extracorporeal shock wave (SW) therapy is an emerging non-invasive physical treatment modality widely applied for tissue repair and inflammation regulation across multiple organs and systems (10, 11). It offers distinct advantages, including non-invasiveness, precise lesion targeting, and adjustable energy levels tailored to specific pathologies (12). Preclinical studies demonstrate that SW recruits endothelial progenitor cells and stimulates angiogenesis in chronic hindlimb ischemia models, thereby enhancing post-ischemic tissue recovery (13, 14). In cardiovascular diseases, SW exhibits anti-inflammatory properties, which mitigate myocardial remodeling and injury during ischemia-reperfusion. SW also upregulates the expression of vascular endothelial growth factor (VEGF), regulates the mRNA expression of endothelial cells, and promotes the formation of therapeutic neovascularization, improving microcirculation to relieve angina pectoris and enhance cardiac function (15, 16). In respiratory research, SW induces the proliferation of bronchial epithelial cells and primary bronchial fibroblasts from patients with chronic obstructive pulmonary disease (COPD), supporting its therapeutic potential in murine models of emphysema (17). Combined SW and mitochondrial therapy reduces pulmonary inflammation and attenuates acute respiratory distress syndrome (ARDS)-associated lung injury (18). The above research collectively highlights that SW has certain therapeutic potential for promoting lung injury repair and cell proliferation.

In this study, lung histopathology, inflammation levels, oxidative stress index, and mitochondrial function were assessed by combining *in vitro* and *in vivo* models. Based on the mechanistic clue that the outward migration of mtDNA mediates the activation of inflammatory corpuscles of NLRP3, which leads to pyroptosis, we explore the molecular mechanism of SW in alleviating sepsis-associated ALI by regulating mitochondrial function and pyroptosis, providing a theoretical basis for clinical transformation.

## 2 Materials and methods

### 2.1 Experimental animals and modeling groups

48-Male C57BL/6N mice (6–7 weeks old, 20 ± 2 g body weight) were purchased from SiPeiFu (Beijing) Biotechnology Co., LTD (Beijing, China; Production License No. SCXK [Beijing] 2024-0001) and housed in a specific pathogen-free (SPF) facility at the Clinical Medical Research Institute of China-Japan Friendship Hospital. Animals were maintained under controlled conditions (20-26°C, 12-hour light/dark cycle) with ad libitum access to food and water. All experimental protocols were approved by the Animal Ethics Committee of China-Japan Friendship Hospital Clinical Medical Research Institute (Approval No.190212).

After a 5-day acclimatization period, the mice were randomly divided into four groups according to their body weight. There were 12 mice in each group, and six mice at each observation point. Control group: Received normal saline via the tail vein without additional intervention. Control + SW group: Received normal saline via the tail vein, followed by SW intervention. LPS group: Received LPS via the tail vein without further treatment. LPS+SW group: Received LPS via the tail vein for 24 h post-modeling, followed by SW intervention.

The septic ALI mouse model was induced by intravenous tail injection of lipopolysaccharide (LPS, 8 mg/kg) (HY-D1056, MCE). The LPS solution was prepared by diluting it with normal saline. First, the mice were fixed and placed in a fixator with their tails exposed. The tails were gently straightened and disinfected with alcohol cotton balls (which also helped remove the keratin and facilitate the visualization of the blood vessels). The tail vein was identified, and red parallel blood vessels could be seen under the light. The injection procedure involved placing the needle tip upwards at an angle of 15-30° to the tail and inserting it into the skin parallel to the blood vessels. The needle was then slightly lifted to enter the vein. After successful insertion, blood return could be observed. Slowly inject the LPS solution, with an injection volume of 100 μL/10 g body weight. After the injection, press the needle hole with a dry cotton ball for 10 s to stop the bleeding, then put it back in the cage for observation for 30 min to ensure there were no abnormal conditions such as bleeding or shock.

**Figure 1:**
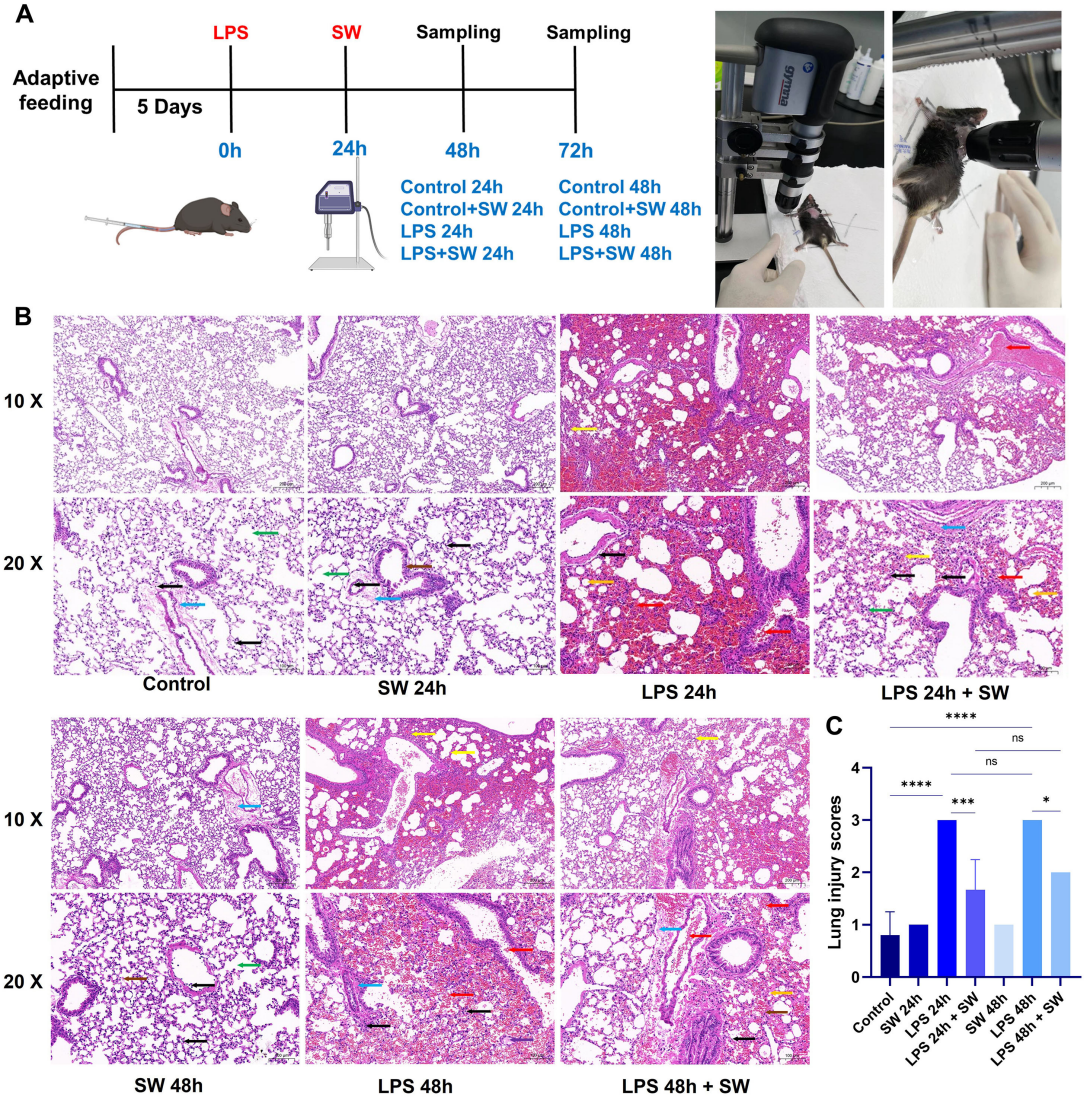
Modelling and histopathological characterization of septic ALI mice. **(A)** Schematic of the experimental timeline and group allocation. **(B)** Representative HE-stained lung sections (scale bar: 50 μm). Pathological features were annotated as follows: Yellow arrows: Alveolar collapse and structural distortion; Red arrows: Alveolar cavity and bronchus haemorrhage and erythrocyte infiltration; Orange arrows: Lipid vacuolization; Black arrows: Interstitial inflammatory cell infiltration; Purple arrows: Proteinaceous exudates in alveolar spaces; Blue arrows: Perivascular fibrosis; Green arrows: AT2 hyperplasia; Brown arrows: Alveolar wall necrosis and nuclear pyknosis. **(C)** Quantitative histopathological scoring of lung injury severity. Data are expressed as mean ± SD and analyzed by One-way ANOVA. n=6. SW, shock wave. ns, P > 0.05; *P < 0.05; ***P < 0.001; ****P < 0.0001.

During the intervention, mice were anaesthetized by intraperitoneal injection of pentobarbital sodium (80 mg/kg), and thoracic hair removal was performed. The mice were fixed on a foam board platform. Ultrasonic coupling gel was applied to optimize energy transmission. After thoroughly applying the coupling agent medium to the chest, the shock wave probe was aimed at the chest and came into contact with the coupling agent medium. The shock wave was delivered using a Gymna Physio ShockMaster 300 system (GymnaUniphy NV, Bilzen, Belgium) with standardized parameters: 0.09 mJ/mm² × 200 impulses at a frequency of 10 Hz. At 24 h and 48 h after SW intervention, mice were euthanized via intraperitoneal injection of sodium pentobarbital (80 mg/kg), followed by tissue harvesting. The grouping strategy, experimental timeline, and shock wave intervention process are summarized in **Figure 1A**.

### 2.2 Sampling scheme

The sampling protocol was the same at different time points. After intraperitoneal injection of pentobarbital sodium (80 mg/kg), the mice’s body weights were recorded. Blood samples were collected from the eyeballs, and after standing for 2 hours at room temperature, the serum was separated by centrifugation at 3000 rpm for 15 min at 4°C. A “T” incision was made from the trachea of the throat, and endotracheal intubation was performed. The thoracic cavity was exposed surgically, the right lung lobe was ligated, and 0.5 mL of precooled saline was injected for bronchoalveolar lavage, repeated 2–3 times, and centrifuged at 1600 rpm for 10min at 4°C. Serum and BALF, stored at -80°C, were used for ELISA and oxidative stress indicator kits. Lung tissues were surgically removed, and 1 mm³ grain-sized tissues from the left lung were fixed with 1% osmium tetroxide in the dark at room temperature and used for electron microscopy. The remaining left lung tissue was fixed in 4% paraformaldehyde, embedded in paraffin, and subjected to pathological examination. Right lung tissues were stored at -80 °C and used for tissue grinding, protein supernatants, and RNA extraction.

### 2.3 Hematoxylin-eosin staining

Lung tissues were fixed in 4% paraformaldehyde for 48 hours, dehydrated through a graded ethanol series, cleared in xylene, and embedded in paraffin. Sections (4 μm thickness) were cut from paraffin blocks, deparaffinized, rehydrated, and stained with hematoxylin and eosin (H&E) using standard protocols. Briefly, sections were stained with hematoxylin for 5 min, differentiated in 1% acid alcohol, counterstained with 0.5% eosin for 2 min, dehydrated, cleared, and mounted with neutral resin. Lung injury was assessed based on the Szapiel scoring system (19). 0 points: No evidence of alveolitis. 1 point: Mild alveolitis, characterized by localized mononuclear cell infiltration near the pleura (<20% of total lung area), and the alveolar structure is generally standard. 2 points: Moderate alveolitis (20%–50% lung involvement), showing diffuse inflammatory cell infiltration. 3 points: Severe alveolitis and fibrosis (>50% lung involvement), with alveolar consolidation due to mononuclear cell accumulation and/or haemorrhage.

### 2.4 Transmission electron microscopy for lung mitochondrial ultrastructural analysis

Lung tissue samples (1 mm³ in size) were fixed in 1% osmium tetroxide prepared in 0.1 M phosphate buffer for 2 hours at room temperature under light-protected conditions. After fixation, tissues were rinsed three times with 0.1 M phosphate buffer (15 min per wash), followed by sequential dehydration in a graded ethanol series (20 min per step) and two washes with 100% acetone (15 min each). Samples were infiltrated with a 1:1 mixture of acetone (10000418, Sinopharm Chemical Reagent Co., Ltd., Cat.) and SPI-812 epoxy resin (90529-77-4, SPI Supplies), then embedded in pure resin and polymerized at 37°C overnight. Polymerization was completed at 60°C for 48 hours. Ultrathin sections (60–80 nm thickness) were cut using an ultramicrotome, collected on 150-mesh Formvar-coated copper grids, and stained with uranyl acetate and lead citrate. Mitochondrial ultrastructure was imaged under a transmission electron microscope (HT7800, Hitachi Ltd., Japan).

### 2.5 Quantification of inflammatory cytokines by ELISA

Inflammatory cytokines of serum, bronchoalveolar lavage fluid (BALF), and cell culture supernatants were detected by ELISA. The content of Tumor necrosis factor-α (TNF-α) (JYM0218Mo, Wuhan Jiyinmei biotech) (EK282/3, LiankeBio), IL-1β (JYM0531Mo, Wuhan Jiyinmei biotech) (EK201B/3, LiankeBio), IL-6 (JYM0012Mo, Wuhan Jiyinmei biotech) (EK206/3, LiankeBio), IL-8 (JYM0457Mo, Wuhan Jiyinmei biotech) was detected according to the kit’s instructions. The universal experimental procedure is as follows: Firstly, the standard was diluted by gradient concentration. Subsequently, add the diluted standard solution and the sample. Then, 50 μL of the enzyme-labeled reagent was added to each well, and the plates were sealed with a plate sealing membrane and incubated at 37°C for 30 min. Dilute 1× detergent with distilled water, wash the plate 5 times, add 50 μL of chromogenic agent A to each well, add 50 μl of chromogenic agent B to each well, gently shake and mix, develop color at 37°C for 10 min in the dark, add 50 μL of termination solution to each well to terminate the reaction. The absorbance of each well was measured at 450nm. The concentration-optical Density (OD) value curve of the standard was calculated. At last, the cytokine concentration of each well was calculated based on this curve.

### 2.6 Analysis of oxidative stress biomarkers

Systematic quantification of redox homeostasis parameters, including superoxide dismutase (SOD) (E-BC-K020-M, Elabscience), glutathione (GSH) (E-BC-K030-M, Elabscience), malondialdehyde (MDA) (E-BC-K025-M, Elabscience), reactive oxygen species (ROS) (CA1410, Solarbio), and myeloperoxidase (MPO) (E-BC-K074-M, Elabscience), was performed in serum and lung tissue homogenates. The experimental procedure was performed according to the kits’ instructions. First, the standards were diluted according to the instructions. The test samples were pre-treated. Corresponding control buffer or test samples were added to the blank tubes and test tubes. Then, the enzyme reaction solutions and reaction color development solutions of different kits were added. After incubation at 37°C, the optical density (OD) value was measured using a microplate reader.

### 2.7 ATP content detection

ATP content in lung tissues and cells was detected by ATP assay kit (BC0305, Solarbio) and following optimized extraction protocols. ATP extraction from lung tissue: according to tissue mass (g), the volume of extraction liquid (mL) is 1:5-10. After centrifugation at 8000 g for 10 min at 4°C, the supernatant was transferred to another EP tube, and 500 μL of chloroform was added to mix thoroughly. After centrifugation at 10000 g for 3 min at 4°C, the supernatant was collected and placed on ice for testing. Cell ATP extraction: Type II alveolar epithelial cells were cultured in 6-well plates at a density of 5 × 10^5 cells per well. The cells were then collected into a centrifuge tube, centrifuged, and the supernatant discarded. According to the number of cells (10^5 cells), the ratio of extraction liquid volume (mL) was 500 to 1000:1, that is, 1 mL of extraction solution was added to the precipitated cells. Cells were sonicated in an ice bath for 1min at 20% intensity or 200W, sonicated for 2s, and stopped for 1s. Centrifugation at 10000g, 4°C for 10min; The supernatant was taken into another EP tube, and 500μL of chloroform was added and thoroughly shaken and mixed. The supernatant was centrifuged at 10000 g, 4°C for 3 min, and the supernatant was removed and placed on ice for testing.

The microplate reader was preheated for more than 30 min, the wavelength was adjusted to 340 nm, and distilled water was set to a baseline of zero. The 10 μmol/mL ATP standard solution was diluted 16 times with distilled water, resulting in a 0.625 μmol/mL standard solution. Add 20 μL of each sample and standard solution to the 96-well UV plate. Add 128 μL of Reagent 1 to each well and 52 μL of the working solution. After thorough mixing, the absorbance value (A1) at 340 nm of the sample was measured immediately for 10 s. Then, the tube containing the reaction solution was placed in a 37°C water bath for 3 min. It was removed, wiped clean, and the absorbance value (A2) was determined immediately, within 3 min and 10 s. The 96-well plates were placed in an incubator at 37°C. ΔA assay =A2 assay tube -A1 assay tube, and ΔA standard =A2 standard tube -A1 standard tube were calculated separately. ATP content (μmol/g mass) was calculated according to the formula = ΔA determination ÷ (ΔA standard ÷C standard concentration) ×V extraction÷ W (sample mass).

### 2.8 qRT-PCR detection of mtDNA expression

Total RNA was isolated from lung tissues and AT2 cells using TRIZOL reagent (H10318, Transgen Biotechnology). RNA was reverse transcribed into cDNA by using the FastKing one-step method to remove the first strand of the genomic cDNA synthesis kit (KR118-02, TIANGEN BIOTECH). Design primers and prepare a reverse transcription reaction system. The template RNA was thawed on ice, and 5× FastKing-RT SuperMix and RNase-Free ddH2O were thawed at room temperature and then quickly placed on ice after thawing. The reverse transcription reactant was prepared with 4 μL of 5×FastKing-RT SuperMix, 50 ng-2 μg of Total RNA, and supplemented to 20 μL with RNase-Free ddH_2_O. The reaction was carried out at 42°C for 15 min to remove the genome and reverse transcription. The reaction at 95°C for 3 min was used to inactivate the enzyme, and reverse transcription was performed. PCR amplification was performed in a fluorescence quantitative PCR instrument (7500, ABI). Initial denaturation was performed at 95°C for 3 min, followed by 40 cycles of 95°C for 30 s and 55°C for 20 s, and finally 72°C for 20 s. qPCR curves were generated, and the Ct values of the samples were recorded. The sequences of the primers used are shown in **Table 1**.

**Table 1:** Primer sequences.

Primers	Sequence
Mus actin_F	GTCCCTCACCCTCCCAAAAG
Mus actin_R	GCTGCCTCAACACCTCAACCC
Mus mtDNA_F	GCCGGTGACTACGACTGAA
Mus mtDNA_R	CACTGGCCTGCAAGTCTTC

### 2.9 Protein expression analysis by western blotting

The total protein from lung tissue and cells was extracted on ice using RIPA lysate (C1053, Applygen Technologies Inc.) and PMSF (ST505, Shanghai Biyuntian Biotechnology). In addition, extract nuclear and cytoplasmic proteins using a nuclear-cytoplasmic fractionation kit (P0028, Shanghai Biyuntian Biotechnology). Total protein concentrations were determined by BCA assay. Samples were mixed with 5× loading buffer and heat denatured at 95°C for 10 min. Add the protein sample into the loading hole of the gel. Electrophoretically separated proteins were transferred onto 0.45 μm PVDF membranes (IPVH00010, Millipore). Membranes were blocked with 5% non-fat milk in TBST for one hour at room temperature. Incubation with primary antibodies: Inducible Nitric Oxide Synthase (iNOS) (1:500, 18985-1-AP, Proteintech), Mitochondrially Encoded NADH Dehydrogenase 2 (MT-ND2) (1:500, 19704-1-AP, Proteintech), Mitochondrially Encoded NADH Dehydrogenase 4 (MT-ND4) (1:500, Ab219822, Abcam), NLRP3 (1:1000, ab263899, Abcam), ASC (1:5000, 10500-1-AP, Proteintech), Caspase 1(1:1000, 83383, CST), GAPDH (1:5000, 60004-1-1g, Proteintech), P65 (1:1000, 8242, CST), LaminB (1:10000, 66095-1-Ig, Proteintech). After overnight incubation of primary antibodies at 4°C, membranes were incubated with HRP-conjugated secondary antibodies corresponding to the species for one hour at room temperature. The membranes were visualized using an ECL luminescence solution (180-501, Shanghai Tanon Life Science), and the bands were detected by an automatic chemiluminescence analyzer (5260 Muti, Shanghai Tanon Life Science). Western blotting original membrane image has been uploaded to **Supplementary Material 1**.

### 2.10 Isolation, culture and modeling of mouse AT2 cells

Fetal mice were euthanized via pentobarbital sodium overdose. Lung tissues were removed under aseptic conditions and placed in a pre-cooled PBS solution. Removed residual connective tissue and washed with PBS solution three times. The lung tissue was cut into pieces, washed once with 0.25% trypsin (containing 0.01% DNase I) solution, and then stirred and digested with 0.25% trypsin (containing 0.01% DNase I) solution at 37°C for 20 min. Digestion was terminated with the same amount of DMEM/F12 (SH300023.01, Hyclone) complete medium supplemented with 10% FBS. The cell suspension was filtered through a 200-mesh sieve and then centrifuged at 1500 rpm for 5 min. The cell precipitate was collected and digested in an incubator with 0.1% collagenase type I at 37°C and 5% CO_2_ for 20 min. After digestion, the cells were terminated with DMEM/F12 complete medium and centrifuged at 1000 rpm for 5 min. The cell precipitate was then collected (20).

The cell suspension was inoculated into a culture bottle and incubated in a 5% CO_2_ incubator at 37°C for 45 min. Most of the adherent cells are fibroblasts. Most of the suspended cells are AT2 cells, along with some fibroblasts and other miscellaneous cells. Suck out the culture solution, inoculate it in another culture bottle, and continue to incubate at 37°C for 40 min, twice in total. Finally, the culture medium was sucked out, centrifuged at 800 rpm for 5 min, and the supernatant was removed. The precipitate cells were seeded at a density of 1 × 10^6 cells/cm^2 in a 10-cm dish and cultured with DMEM/F12 complete medium containing 10% FBS for 24 h at 37°C in a 5% CO_2_ incubator. Then, change the culture medium to remove the cells that are not attached to the wall. Observe the growth state and morphological characteristics of cells with an inverted microscope.

AT2 cells in good condition were collected into 10 sterilized 5 mL polypropylene tubes; one tube served as a blank control, and nine tubes of cells were intervened with SW using different intervention parameters to reduce crossover effects. After intervention, the cells were seeded in 96-well plates (100 μL/well, 4 × 10^3 cells/well) and incubated for 24 h at 37°C in a 5% CO_2_ incubator. Untreated AT2 cells were used as control, and different intervention parameters of SW were screened: 1.0 Bar ×200 impulses with 1 Hz; 1.0 Bar ×500 impulses with 1 Hz; 1.0 Bar ×800 impulses with 1 Hz; 1.5 Bar ×200 impulses with 1 Hz; 1.5 Bar ×500 impulses with 1 Hz; 1.5 Bar ×800 impulses with 1 Hz; 2.0 Bar ×200 impulses with 1 Hz; 2.0 Bar ×500 impulses with 1 Hz; 2.0 Bar ×800 impulses with 1 Hz. After 24 hours of intervention, 10 μL CCK-8 reagent (CK04, DoJinDo) was added to each well and incubated at 37°C for 2 hours. The absorbance value was measured at 450 nm using a microplate reader. The maximum intervention parameters screened to avoid affecting AT2 activity were 500 impulses at a frequency of 1 Hz, with an energy intensity of 1.5 Bar. (CCK-8 results are presented in **Supplementary Material 2**).

The cells were divided into three groups according to different intervention methods. The Control group was cultured routinely without any treatment. In the LPS+ATP group, AT2 cells were treated with LPS (HY-D1056, MCE) at a concentration of 100ng/ml for 24h, followed by ATP (HY-B2176, MCE) at a concentration of 5mM for 30min. The pyroptosis model was induced by stimulation with LPS and ATP (21). In the LPS+ATP+SW group, based on the pyroptosis model, the culture medium was changed, and AT2 cells were collected into sterile 5 mL polypropylene tubes. The bottom of the tube was intervened with dispersive extracorporeal SW (Aries 2, German Dornier), and the cells were seeded and cultured for 24 hours after intervention. Finally, the cells were digested and collected for subsequent index detection (11).

### 2.11 Assessment of mitochondrial membrane potential using JC-1 fluorometric assay

JC-1 is a fluorescent probe that can penetrate organelles and bind explicitly to mitochondria in cells. Changes in the red and green fluorescence colours can detect the changes in mitochondrial membrane potential. Follow the instructions of the mitochondrial membrane potential detection kit (JC-1) (M8650, Solarbio). Dilute JC-1 according to the ratio of 50 μL JC-1 (200 ×) for every 8 mL of ultrapure water. Vigorously shake to dissolve and thoroughly mix JC-1. Then, add 2 mL of JC-1 staining buffer (5×) and mix well to obtain the JC-1 staining working solution. The cells were inoculated into 6-well plates and treated according to different intervention methods. The cells were washed once with PBS buffer solution, and the culture medium was removed. 1 mL of culture medium and 1 mL of JC-1 staining working solution were added to each well, and they were thoroughly mixed. The cells were incubated at 37°C in a 5% CO_2_ incubator for 20 min. During the incubation period, prepare an appropriate amount of JC-1 staining buffer (1×) by adding 4 mL of distilled water for every 1 mL of JC-1 staining buffer (5×). Store the 1× JC-1 staining buffer on ice. After incubation, the culture medium was aspirated, and the cells were washed twice with JC-1 staining buffer (1×). Then, 1 mL of culture medium was added, and the sample was observed under a fluorescence microscope (IX73, Olympus).

### 2.12 Assessment of mitochondria superoxide by MitoSOX probe

The cells were inoculated into 12-well plates at 2 × 10^4 cells per well and allowed to adhere to the surface. Then, they were processed according to different groups. Dilute the serum-free medium to prepare a working solution of 5 μM MitoSOX reagent (M36008, Invitrogen). Remove the culture medium, add 0.5 milliliters of 5 μM MitoSOX working solution to each well, and cover the cells. The cells were incubated at 37°C with 5% CO_2_ in the dark for 30 min. Gently wash the cells three times with PBS buffer to remove the MitoSOX working solution that did not enter the cells. A laser confocal microscope (TCS SP8, Leica) was used to capture fluorescence images.

### 2.13 Intracellular ROS measurement via DCFH-DA fluorogenic probe

The cells were seeded into 12-well plates at 2 × 10^4 cells per well and processed according to the different groups. According to the ROS detection kit (S0033S, Shanghai Biyuntian Biotechnology), DCFH-DA was diluted with serum-free culture solution at a 1:1000 ratio. The cells were then washed once with PBS to remove serum from the medium. Diluted DCFH-DA was added to the cells to immerse them completely. The cells were incubated at 37°C with 5% CO_2_ in the dark for 20 min. Then, the cells were washed three times with serum-free cell culture medium to remove DCFH-DA that did not enter the cells. A laser confocal microscope (TCS SP8, Leica) was used to take fluorescence photos.

### 2.14 Statistical analysis

Use GraphPad Prism 9.0 for statistical analysis and drawing. One-way analysis of variance (ANOVA) was used to compare the groups, followed by Tukey’s *post hoc* test. P < 0.05 was statistically significant. The data is expressed as mean ± SD. All *in vitro* experiments were repeated three times, with three replicates for each experiment.

## 3 Result

### 3.1 SW ameliorates lung injury histopathology in septic ALI mice

HE staining was used to observe the pathological changes of lung tissue at different time points 24 and 48 hours after modelling (**Figure 1B**). In the control group, the lung tissue structure was standard, with no significant haemorrhage, perivascular fibrosis, or inflammatory cell infiltration in the alveolar interstitium. In the LPS-24h and LPS-48h groups, LPS induced marked pathological alterations, including alveolar collapse, thickened alveolar walls, and extensive erythrocyte infiltration within alveolar spaces and terminal bronchioles. In addition, lipid vacuoles, proteinaceous exudates in alveoli, perivascular fibrosis, and interstitial inflammatory cell infiltration. After LPS+ SW intervention for 24h and 48h, SW intervention attenuated LPS-induced damage, characterized by reduced alveolar erythrocyte infiltration, fewer lipid vacuoles, and diminished perivascular fibrosis. AT2 cell and focal alveolar wall were slight hyperplasia, with residual mild interstitial inflammation. In the Control+SW group, minor alveolar atrophy and occasional alveolar fusion were noted without overt haemorrhage. Scant perivascular fibrosis and inflammatory cells were present in the alveolar interstitium and vasculature.

The severity of lung injury was scored to quantitative histopathological assessment. The LPS-24h and LPS-48h groups exhibited markedly higher pathological scores compared to the control group (P < 0.001). Compared with the LPS-24h and LPS-48h groups, the pathological scores of lung tissue were significantly decreased at 24h and 48h after SW intervention (P < 0.05) (**Figure 1C**). The pathological results suggested that the LPS-induced septic ALI mice model was successfully established, and SW could improve the pathological injury of ALI. Notably, the mice receiving SW alone (Control+SW group) showed only mild abnormalities in lung tissue structure, confirming the safety profile of low-dose SW therapy within the tested parameters.

### 3.2 SW regulates inflammatory cytokines and oxidative stress markers in septic ALI mice

**Figure 2:**
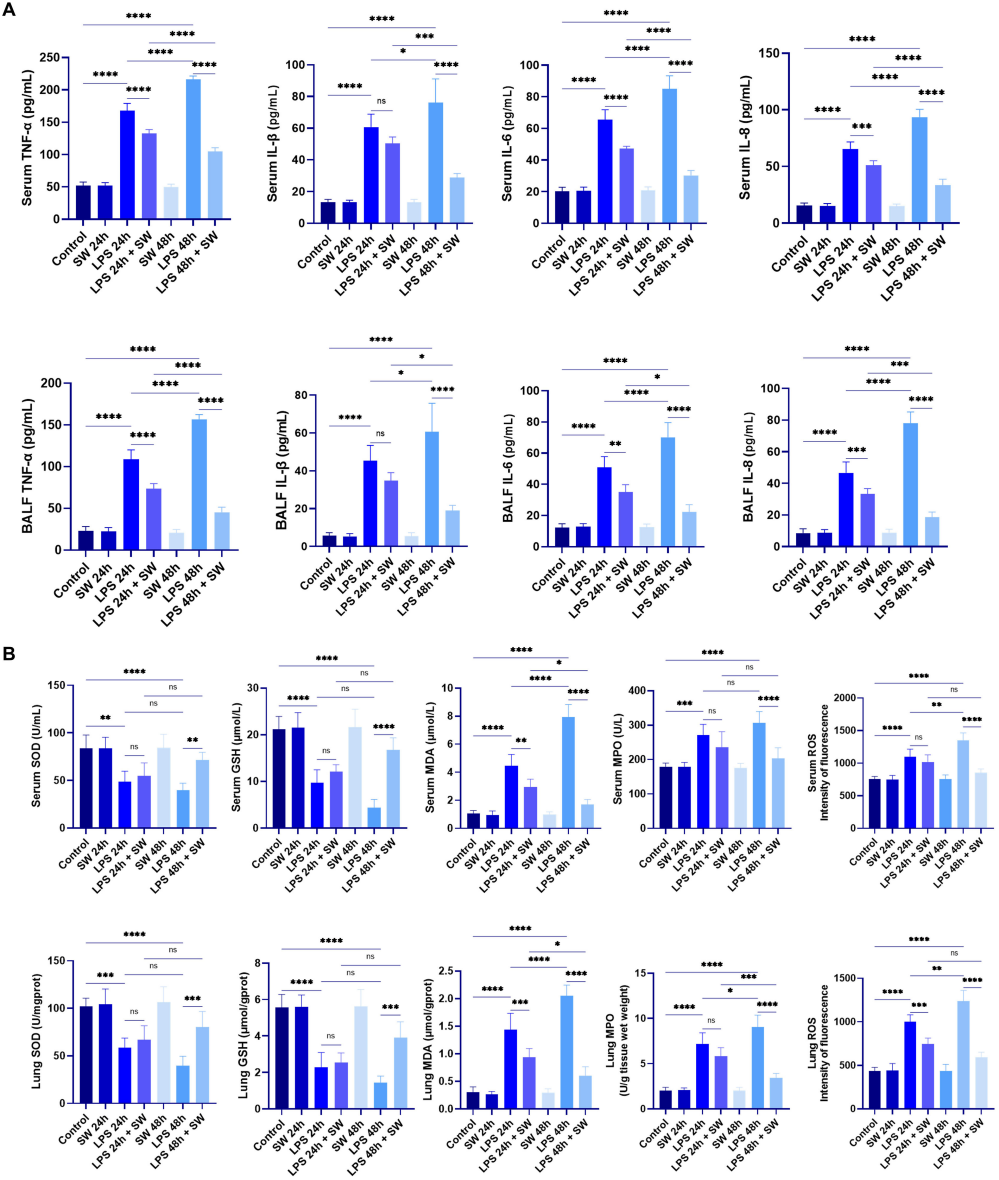
Levels of inflammatory cytokines and oxidative stress in septic ALI mice. **(A)** Secretion levels of inflammatory factors TNF-a, IL-1β, IL-6 and IL-8 in serum and BALF. **(B)** Contents of SOD, GSH, MDA, MPO and ROS in serum and lung tissue grinding fluid. Data are expressed as mean ± SD and and differences among groups were evaluated with one-way ANOVA with Tukey’s *post hoc* test. n=6. BALF, bronchoalveolar lavage fluid; SW, shock wave; TNF-α, Tumor necrosis factor-α; SOD, superoxide dismutase; GSH, glutathione; MDA, malondialdehyde; ROS, reactive oxygen species; MPO, myeloperoxidase. ns, P > 0.05; *P < 0.05; **P < 0.01; ***P < 0.001; ****P < 0.0001.

Serum and BALF concentrations of proinflammatory cytokines (TNF-α, IL-1β, IL-6, and IL-8) were quantified using ELISA. Compared with the control group, the LPS-induced septic ALI mice model demonstrated marked upregulation of all measured cytokines (P < 0.001), with significantly higher concentrations observed at 48h compared to 24h timepoint (P < 0.05). SW intervention significantly downregulated the cytokine levels at both 24h and 48h post-treatment intervals compared to the LPS groups (P < 0.001). Notably, the secretion level of inflammatory cytokines decreased more significantly in the 48h-SW group (P < 0.05) (**Figure 2A**). These findings collectively demonstrate that SW effectively downregulates systemic and pulmonary inflammatory mediators in septic ALI mice.

To assess redox homeostasis, oxidative stress markers, including superoxide dismutase (SOD), glutathione (GSH), malondialdehyde (MDA), myeloperoxidase (MPO), and ROS, were quantified in both serum and lung tissue homogenates. The endogenous antioxidants SOD and GSH exhibited significant depletion in LPS groups compared to the control group (P < 0.01). SW intervention upregulated the SOD and GSH levels compared to LPS groups (P < 0.001). Conversely, oxidative damage biomarkers (MDA, MPO, ROS) demonstrated marked elevation in LPS groups (P < 0.001), which were substantially decreased in the LPS+48h SW group (P < 0.0001) (**Figure 2B**). These data demonstrate that SW could regulate oxidative stress disorder in septic ALI mice.

### 3.3 SW improves mitochondrial damage and function in septic ALI mice

**Figure 3:**
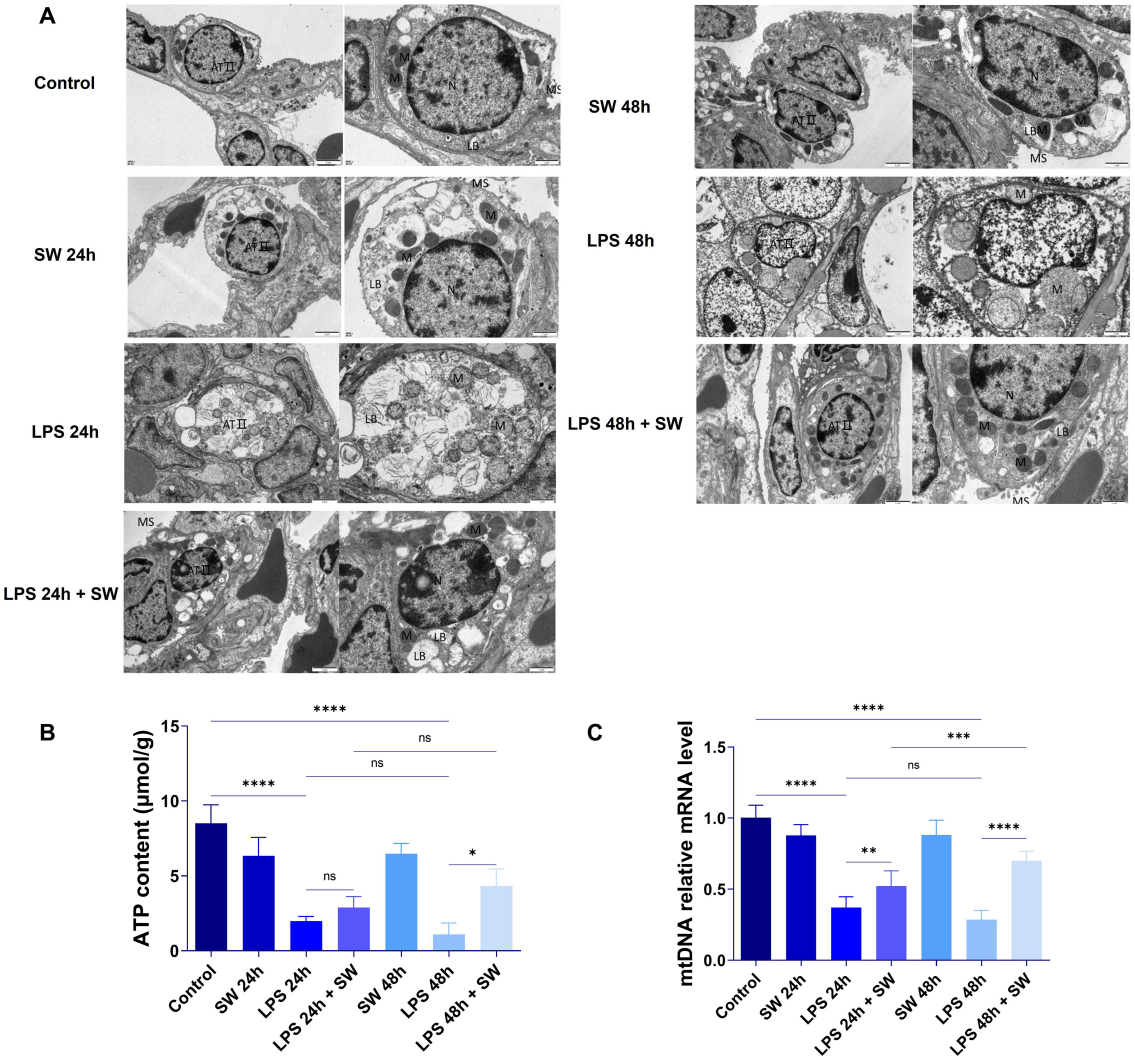
Ultrastructure and mitochondrial function of lung tissue in septic ALI mice. **(A)** Ultrastructure of lung tissue was observed by transmission electron microscope. AT2 cells (AT II), nucleus (N), mitochondria (M), lamellar bodies (LB) and microvilli (MS). **(B)** The changes of ATP in lung tissue were determined by colourimetry; **(C)** Changes of mRNA level in lung tissue mtDNA. Data are expressed as mean ± SD and and differences among groups were evaluated with one-way ANOVA with Tukey’s *post hoc* test. n=6. mtDNA, mitochondrial DNA; SW, shock wave. ns, P > 0.05; *P < 0.05; **P < 0.01; ***P < 0.001; ****P < 0.0001.

Mitochondrial damage is the initial factor of sepsis-related ALI. The microstructure and morphological changes of mitochondria in lung tissue were observed by transmission electron microscope. In the control group, the pulmonary capillary loops demonstrated orderly arrangement, with AT2 cells maintaining regular morphology. These cells contained ovoid nuclei (N) and well-preserved cytoplasmic organelles, including mitochondria (M) displaying distinct cristae and intact membranes. Lamellar bodies (LB) maintained structural integrity, and short microvilli (MS) were consistently observed on cellular surfaces. These features remained stable in both 24h and 48h control groups. The LPS groups exhibited time-dependent pathological progression. In the LPS-24h group, pulmonary architecture showed capillary loop disorganization and ATII cellular edema. Mitochondria displayed cristae fragmentation and partial membrane disruption, accompanied by LB swelling and microvilli loss. The LPS-48h group manifested exacerbated cellular damage, featuring marked organelle degeneration and mitochondrial vacuolization. Notably, mitochondrial swelling intensity correlated with LPS exposure duration, indicating progressive ultrastructural deterioration. In the LPS+SW24h and LPS+SW48h group, the pulmonary capillary loops were irregularly arranged; AT2 cells have regular morphology, a slightly irregular nucleus (N), oval or short rod-shaped mitochondria (M) in the cytoplasm, slightly blurred inner ridge of mitochondria, clear structure of lamellar bodies (LB) in the cytoplasm and short microvilli (MS) on the cell surface. Compared with the LPS+SW 24 h group, the abnormality of cells and organelles in the LPS+SW 48 h group is lighter, the cell morphology is regular, microvilli exist, the mitochondria morphology is normal, the outer membrane is intact, and the inner ridge is clear (**Figure 3A**).

Sepsis-induced mitochondrial dysfunction is characterized by organelle damage and impaired ATP synthesis. The transfer and release of mtDNA from tissues and cells to cells is the key link of sepsis-related ALI. The results showed that compared with the control group, the ATP content in the lung tissue of mice was significantly decreased after LPS modeling, suggesting that the mitochondrial function of mice was damaged during sepsis. However, the ATP level of lung tissue in the LPS-SW 24 h group showed an upward trend, and the ATP level in the LPS-SW 48 h group was significantly increased (P < 0.05) (**Figure 3B**). Q-PCR detected the expression of mtDNA in lung tissue. Compared with the control group, the mRNA expression of mtDNA decreased significantly after LPS modeling. However, the mRNA expression of mtDNA in the LPS+SW24h and 48h groups increased significantly (P < 0.01), and the up-regulation of mRNA in the LPS+SW48h group was more significant (P < 0.001) (**Figure 3C**). The above that SW is helpful to the recovery of mitochondrial function of ALI related to sepsis in a mouse sepsis model. These results demonstrate that SW therapy significantly enhances the recovery of mitochondrial function in the lung tissue of septic ALI mice. Notably, the 48h SW intervention shows a superior repair treatment effect.

### 3.4 SW regulates the signal pathway of mtDNA-mediated pyroptosis

**Figure 4:**
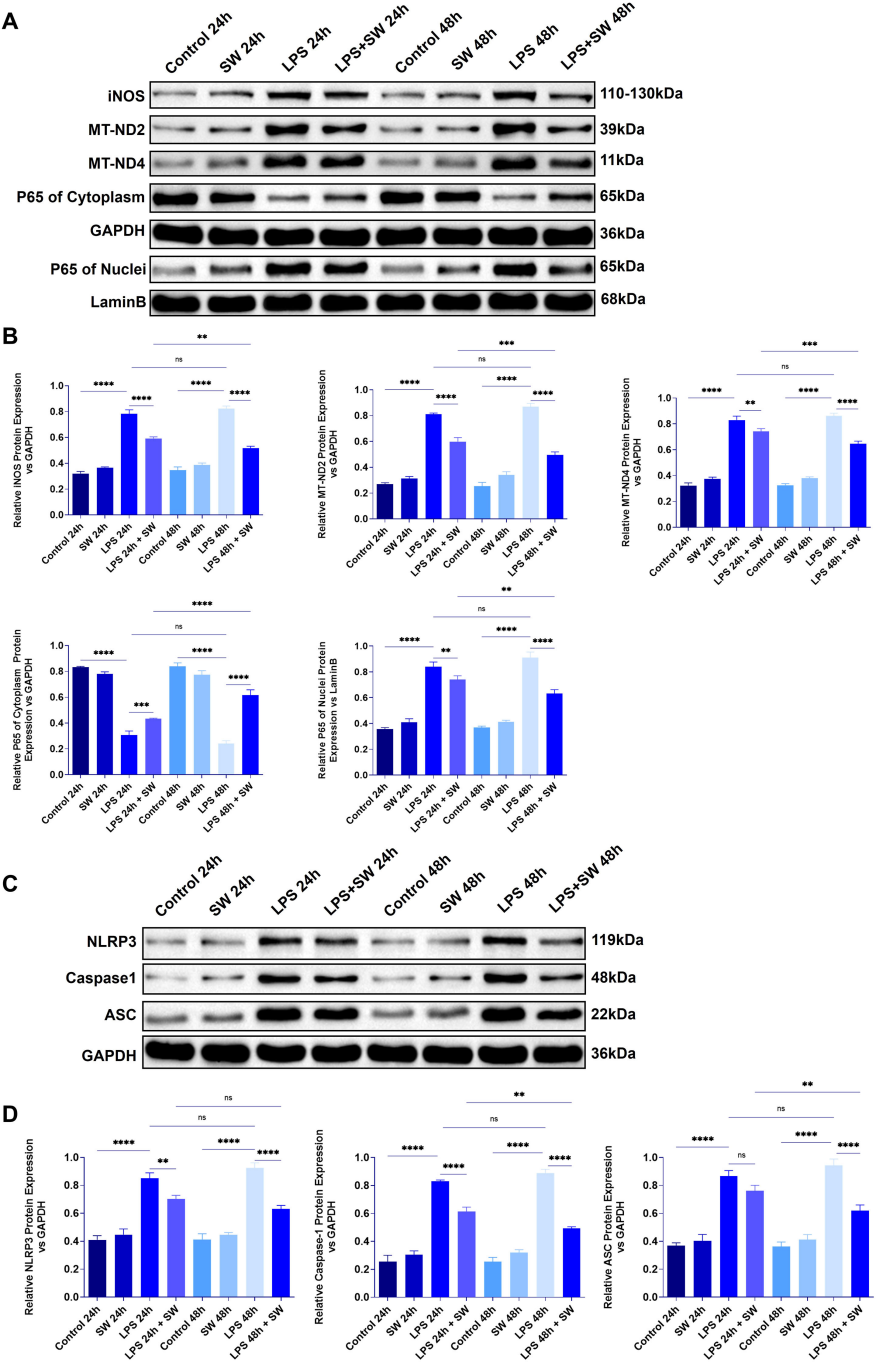
Expression of mitochondrial damage and pyroptosis-related proteins in lung tissue of septic ALI mice **(A)** The protein expression of iNOS, MT-ND2, MT-ND4, and p65 in the cytoplasm and cytoplasm of lung tissue were detected. **(B)** Statistical analysis of iNOS, MT-ND2, MT-ND4, and p65 protein relative expression in cytoplasm and cytoplasm. **(C)** The protein expression of NLRP3, ASC and Caspase-1 in lung tissue. **(D)** Statistical analysis of the relative expression of NLRP 3, ASC and Caspase-1. Data are expressed as mean ± SD and and differences among groups were evaluated with one-way ANOVA with Tukey’s *post hoc* test. n=3. iNOS, Nitric Oxide Synthase; MT-ND2, Mitochondrially Encoded NADH Dehydrogenase 2; MT-ND4, Mitochondrially Encoded NADH Dehydrogenase 4; SW, shock wave. ns, P > 0.05; *P < 0.05; **P < 0.01; ***P < 0.001; ****P < 0.0001.

After mitochondrial damage in sepsis, mtDNA is released, which amplifies inflammatory reactions. We detected proteins related to mitochondrial damage and pyroptosis in the lung tissue of septic ALI mice to explore the mechanism of SW. Compared with the control group, after LPS modeling, p65 was translocated into the nucleus, and the protein expressions of iNOS, MT-ND2, and MT-MD4 were significantly increased (P < 0.0001), indicating that the LPS-TLR4 signal drove NF-κB activation, leading to p65 translocation and promoting the production of iNOS. The function of the mitochondrial respiratory chain, especially complex I, is damaged, and the expressions of MT-ND2 and MT-ND4 are upregulated, leading to a decrease in oxidative phosphorylation efficiency and ATP levels. However, in the LPS+SW24 and LPS+SW48h groups, the translocation of the p65 nucleus decreased, the protein expressions of iNOS, MT-ND2, and MT-MD4 were significantly decreased (P < 0.01), and the intervention effect of LPS+SW48h group was more significant (P < 0.01) (**Figures 4A, B**).

Mitochondrial membrane rupture leads to the leakage of mtDNA into the cytoplasm or the circulatory system. As the receptor of damage-related molecular patterns (DAMPs), mtDNA activates the inflammatory corpuscles of NLRP3. Compared with the control group, the expressions of NLRP3, ASC, and Caspase-1 in lung tissue after LPS modeling were significantly increased (P < 0.0001), while the expressions of NLRP3, ASC, and Caspase-1 in LPS+SW24 and 48h groups were significantly decreased (P<0.0001), especially in LPS+SW48h group (**Figures 4C, D**). The above results show that the mechanism of SW is related to improving mitochondrial damage and the process of pyroptosis.

### 3.5 SW improves mitochondrial damage in the AT2 cell pyroptosis model

**Figure 5:**
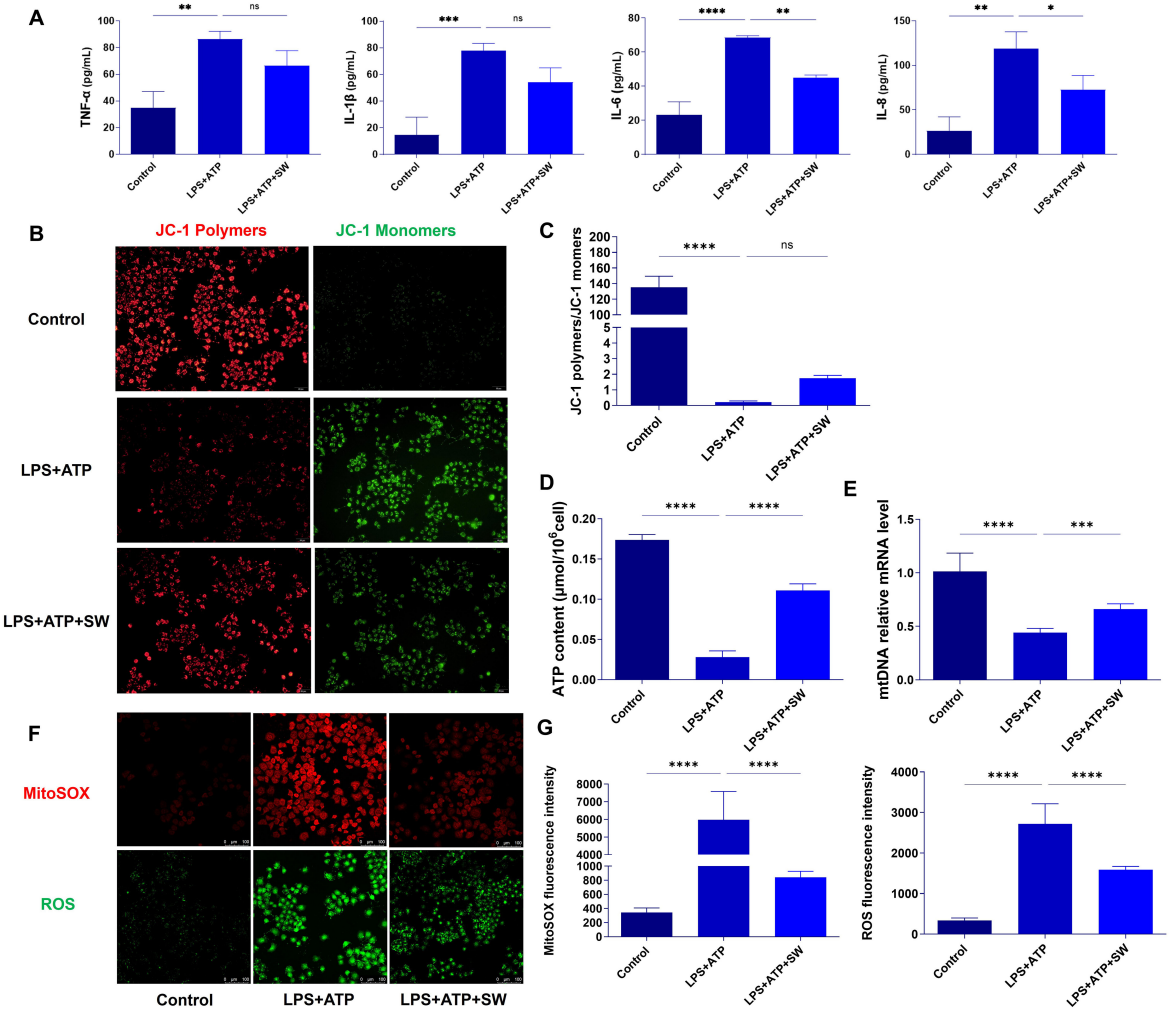
SW improves mitochondrial damage in AT2 cell pyroptosis model **(A)** The expression levels of TNF-α, IL-1β, IL-6 and IL-8 in type II alveolar epithelial cells were detected by ELISA. **(B)** JC-1 fluorescence probe method was used to observe the mitochondrial membrane potential, with polymer on the left (red fluorescence) and monomer on the right (green fluorescence). **(C)** The ratio of JC-1 polymer to JC-1 monomer, that is, the ratio of red fluorescence to green fluorescence, the higher the ratio, the higher the membrane potential. **(D)** Q-PCR detected the expression of mtDNA. **(E)** The content of ATP was detected by colourimetry. **(F)** Red fluorescence refers to the MitoSOX level, and green fluorescence refers to the ROS level. **(G)** Statistics of fluorescence intensity of MitoSOX and ROS. Data are expressed as mean ± SD and and differences among groups were evaluated with one-way ANOVA with Tukey’s *post hoc* test. n=3. The experiment was repeated three times. mtDNA, mitochondrial DNA; TNF-α, Tumor necrosis factor-α; SW, shock wave. ns, P > 0.05; *P < 0.05; **P < 0.01; ***P < 0.001; ****P < 0.0001.

The AT2 cell pyroptosis model was established through stimulation with LPS and ATP. The secretion of TNF-α, IL-1β, IL-6, and IL-8 in AT2 cells was detected by ELISA. Compared with the control group, the secretion levels of the aforementioned inflammatory cytokines in the LPS+ATP group were significantly increased (P < 0.01). After SW intervention, the secretion level of inflammatory cytokines decreased (P < 0.01). It is suggested that SW can regulate the level of the inflammatory cytokine in the AT2 cell pyroptosis model (**Figure 5A**).

The hue of the JC-1 fluorescent probe reflects the magnitude of the mitochondrial membrane potential. When the mitochondrial membrane potential is relatively high, JC-1 accumulates within the mitochondrial matrix, forming aggregates that emit red fluorescence. Conversely, when the mitochondrial membrane potential is relatively low, JC-1 fails to accumulate in the mitochondrial matrix and exists in a monomeric form, emitting green fluorescence. The ratio of red fluorescence to green fluorescence in a cell population reflects the overall mitochondrial membrane potential levels among different groups. A higher ratio indicates a higher mitochondrial membrane potential and vice versa. In the control group, the mitochondrial membrane potential was relatively high. JC-1 accumulated in the mitochondrial matrix, with red fluorescence being highly expressed and green fluorescence being lowly expressed. However, JC-1 was unable to accumulate in the mitochondrial matrix in the LPS + ATP group. Green fluorescence was highly expressed, and the ratio of the mitochondrial fluorescence intensity decreased significantly (P < 0.0001), suggesting a decrease in the mitochondrial membrane potential in the AT2 cell pyroptosis model. Compared with the LPS + ATP group, after SW intervention, the ratio of the mitochondrial fluorescence intensity exhibited an upward trend. It indicates that SW can alleviate the decline in the mitochondrial membrane potential in the AT2 cell pyroptosis model (**Figures 5B, C**). Compared with the control group, the intracellular ATP and mtDNA levels in the LPS+ATP group decreased significantly (P < 0.0001). Following SW intervention, the levels of ATP and mtDNA increased significantly (P < 0.001). Most ATP in cells is produced by mitochondria (**Figures 5D, E**). Combined with the fluorescence experiment results of JC-1, it is suggested that SW can improve the mitochondrial function of AT2 cells, promote the synthesis of mtDNA, and regulate the mitochondrial energy imbalance.

To further investigate the regulation of mitochondrial oxidative stress by SW, the level of MitoSOX in mitochondria was detected by the MitoSOX probe (red fluorescence), and the level of intracellular ROS was detected by fluorescent probe DCFH-DA (green fluorescence). Compared with the control group, the fluorescence intensities of both MitoSOX and ROS were significantly higher in the LPS+ATP group (P < 0.0001). However, after SW intervention, the fluorescence intensity of MitoSOX and ROS was significantly lower than that of the LPS+ATP group (P < 0.0001) (**Figures 5F, G**). It is suggested that SW can regulate the imbalance of mitochondrial oxidative stress caused by pyroptosis.

### 3.6 Mechanism of SW in protecting mitochondrial damage and inhibiting pyroptosis

**Figure 6:**
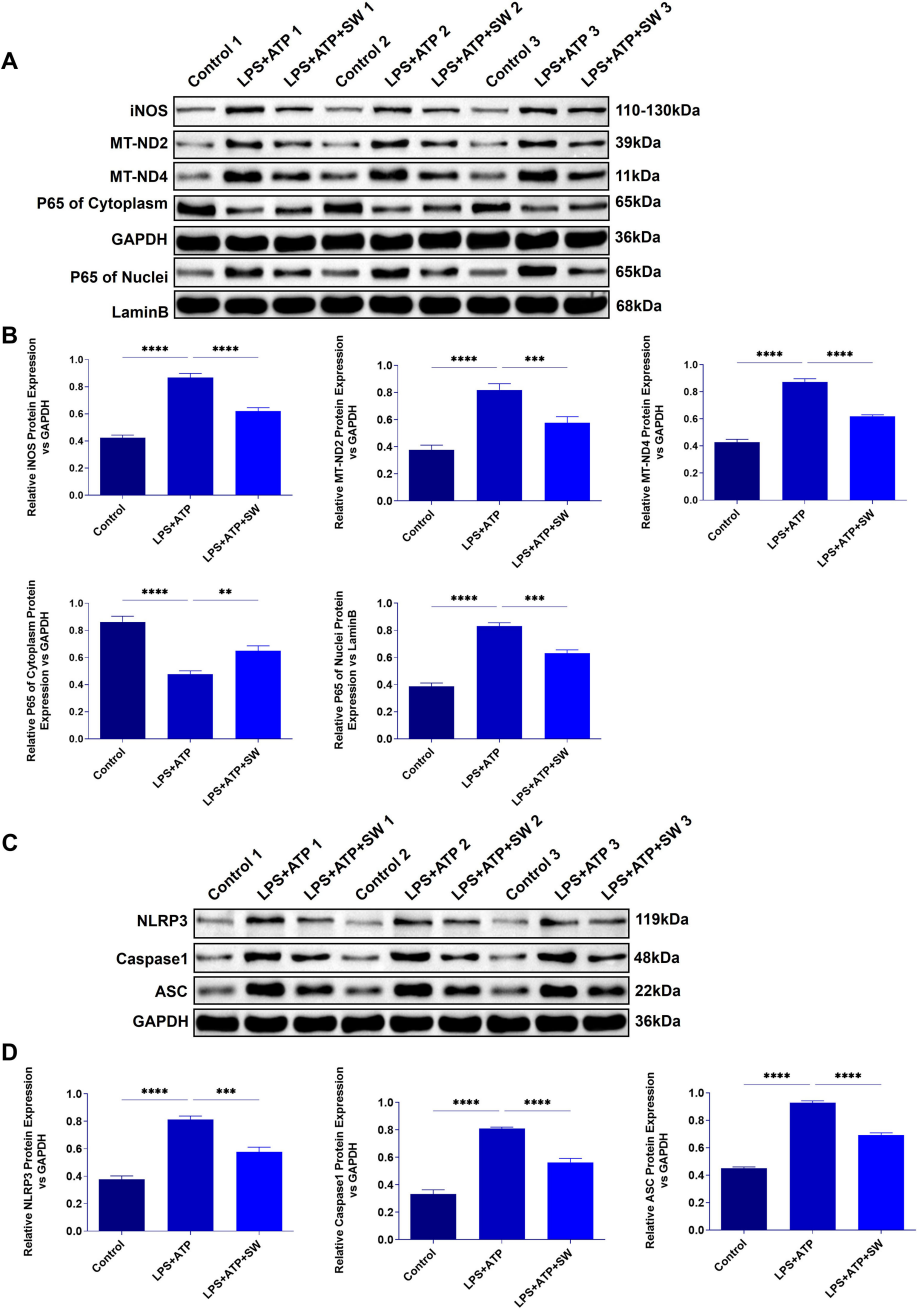
Expression of mitochondrial damage and pyroptosis proteins in AT2 cell model. **(A)** Expression of iNOS, MT-ND2, MT-ND4, and p65 protein in cytoplasm and cytoplasm in AT2 cell model. **(B)** Statistical analysis of iNOS, MT-ND2, MT-ND4, and p65 protein relative expression in cytoplasm and cytoplasm. **(C)** Protein expression of NLRP3, ASC and Caspase-1. **(D)** Statistical analysis of the relative expression of NLRP 3, ASC and Caspase-1. Data are expressed as mean ± SD and and differences among groups were evaluated with one-way ANOVA with Tukey’s *post hoc* test. n=3. iNOS, Nitric Oxide Synthase; MT-ND2, Mitochondrially Encoded NADH Dehydrogenase 2; MT-ND4, Mitochondrially Encoded NADH Dehydrogenase 4; SW, shock wave. **P < 0.01; ***P < 0.001; ****P < 0.0001.

*In vitro* experiments verify the mechanism by which SW regulates the interaction between mitochondria and pyroptosis. Compared with the control group, in LPS+ATP group shows that p65 was translocated and highly expressed in the nucleus, the protein expressions of mitochondrial damage markers (iNOS, MT-ND2 and MT-MD4) were significantly increased (P<0.0001) (**Figure 6A, B**), and the expressions of activated NLRP3 and its downstream ASC and Caspase-1 were significantly increased (P<0.001) (**Figure 6C, D**), suggesting that LPS+ATP could induce mitochondrial dysfunction, oxidative stress imbalance and AT2 cells pyroptosis. However, after SW intervention, the translocation of the p65 nucleus decreased, and the protein expressions of iNOS, MT-ND2, MT-MD4, NLRP3, ASC, and Caspase-1 decreased significantly (P<0.01), indicating that SW intervention can protect mitochondria damage, inhibit the activation of NLRP3 inflammasome and attenuate pyroptosis. In short, it demonstrates the therapeutic potential of SW in disrupting the vicious circle of mitochondrial damage and inflammation amplification in sepsis-related ALI.

## 4 Discussion

**Figure 7:**
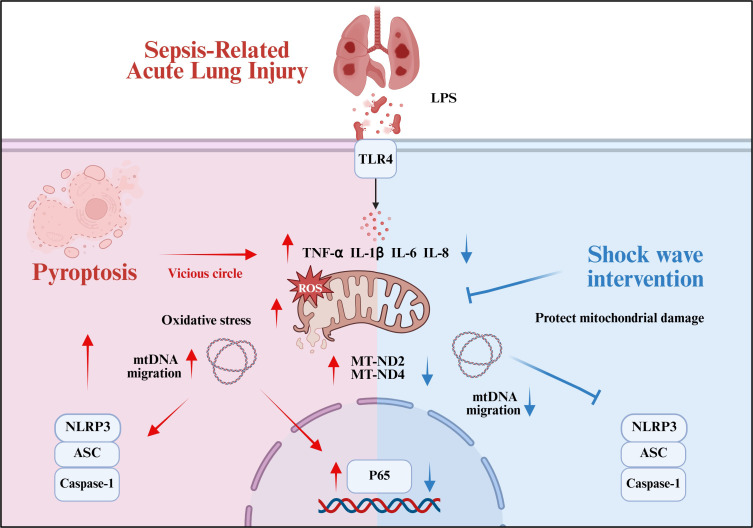
Summary of the mechanism by which SW regulates septic ALI (Created in BioRender. (2025) https://BioRender.com/0f2m7md).

With the continuous development of SW in the biomedical field, its application domains have progressively expanded and diversified. SW demonstrates innovative therapeutic applications across multiple organ systems, including cardiovascular, renal, and pulmonary pathologies. Emerging evidence suggests that SW intervention within appropriate energy parameters can safely enhance organ functional recovery through multiple pathways, promoting angiogenesis and tissue repair while attenuating inflammatory responses, oxidative stress, and apoptotic processes (15, 22–25). It is found that the mechanical energy of shock wave leads to cytoskeletal remodeling, facilitating nuclear-to-cytoplasmic mRNA transport and subsequent activation of subcellular organelles, including mitochondria, endoplasmic reticulum, and vesicular compartments. This cascade modulates inflammatory pathways and stimulates the secretion of regenerative mediators, such as vascular endothelial growth factor (12, 26). In respiratory disease models, SW therapy demonstrates therapeutic potential for COPD and ARDS (17, 18). Our *in vivo* and *in vitro* studies have found that SW therapy ameliorated histopathological alterations in septic ALI mice. The therapeutic mechanism involves preserving mitochondrial functional integrity in AT2 cells, which inhibits NLRP3 inflammasome activation mediated by aberrant mtDNA release. This intervention interrupts the pathological feedback loop of mitochondrial dysfunction-induced pyroptosis cascades (**Figure 7**).

During the progression of sepsis, mitochondrial dysfunction serves not only as a trigger for inflammatory cascades but also as a critical driver of organ dysfunction. Pathogen-associated molecular patterns (PAMPs, e.g., LPS) and inflammatory mediators (TNF-α, IL-1β) induce mitochondrial membrane potential collapse, ROS overproduction, and calcium overload, culminating in the structural disintegration of mitochondrial architecture (3). Damaged mitochondria release oxidatively mtDNA into the cytoplasm and systemic circulation. Elevated circulating mtDNA levels in sepsis patients correlate positively with the severity and prognosis of ALI and ARDS (27–29). In our study, we observed a significant upregulation of proinflammatory cytokines (TNF-α, IL-1β, IL-6, and IL-8) and an imbalance of oxidative stress in septic ALI mice. AT2 cells exhibited severe ultrastructural damage, including mitochondrial fragmentation and disruption of lamellar bodies. Abnormal mitochondrial membrane potential, increased ROS levels, and elevated mitochondrial membrane permeability. Concurrently, ATP content and mtDNA levels were significantly decreased in lung tissues and AT2 cells. However, the SW intervention significantly attenuated cytokine storm-mediated inflammatory injury in ALI mice. Prolonged SW 48h-treatment demonstrated enhanced therapeutic effects: histopathological analysis revealed improved lung and mitochondrial ultrastructure injury. SW intervention could regulate oxidative stress disorder, up-regulate the expression levels of ATP and mtDNA in lung tissue and cells, and protect the mitochondrial membrane potential. These findings indicate that SW therapy ameliorates sepsis-related ALI by preserving mitochondrial functional integrity in AT2 cells.

Mitochondrial integrity is intrinsically linked to alveolar surfactant secretion and the repair of alveolar epithelial and endothelial barriers (30, 31). Mitochondrial function is dually regulated by mitochondrial DNA (mtDNA) and nuclear DNA (nDNA), with mtDNA release acting as a damage-associated molecular pattern (DAMPs) upon mitochondrial membrane rupture. These DAMPs bind pattern recognition receptors (PRRs, e.g., TLR9 and cGAS-STING), activating the NLRP3 inflammasome and triggering pyroptosis in macrophages, alveolar epithelial cells, and endothelial cells (32). In the LPS/ATP-induced pyroptotic AT2 cell model, we found that mitochondrial ROS and membrane permeability were significantly increased, inducing mtDNA release into the cytoplasm. During pyroptosis, the LPS and ATP activate NLRP3 and downstream caspase-1. Upon activation, caspase-1 cleaves Gasdermin D (GSDMD) into its active form, generating an N-terminal fragment (GSDMD-NT). GSDMD-NT binds to acidic phospholipids on the inner leaflet of the cell membrane and oligomerize to form membrane pores, leading to membrane disruption, cellular content leakage, pyroptosis, and the release of proinflammatory cytokines (33). Before pore formation in the plasma membrane, GSDMD-NT translocates to mitochondria. It is binding to cardiolipin induces mitochondrial damage, resulting in disruption of the inner and outer mitochondrial membranes, mitochondrial depletion, enhanced mitophagy, increased ROS production, loss of transmembrane potential, and impaired oxidative phosphorylation. Concurrently, mtDNA is released from the mitochondrial matrix and intermembrane space. The mitochondrial damage releases signaling molecules such as mtDNA and ROS, which activate the NLRP3 inflammasome. This further promotes GSDMD activation and pyroptosis, amplifying the inflammatory cascade and elevating IL-1β and IL-18 secretion (21, 34). Ultimately, the “mitochondrial damage–inflammatory amplification–reinjury” vicious cycle exacerbates ALI/ARDS progression. Notably, the FDA-approved drug disulfiram has been identified as a potent inhibitor of GSDMD pore formation, effectively suppressing pyroptosis and inflammatory cytokine release (35). It suggests GSDMD as a promising therapeutic target for interrupting the mitochondrial damage and inflammatory cascade driven by pyroptosis in sepsis-related ALI. While SW has demonstrated protective effects against mitochondrial damage and mtDNA leakage, its potential role in upstream inhibition of GSDMD pore formation requires further investigation.

Furthermore, our findings revealed marked upregulation of iNOS in lung tissues of LPS-induced septic ALI mice, accompanied by impaired mitochondrial respiratory chain function (particularly Complex I), as evidenced by increased expression of MT-ND2 and MT-ND4. These results confirm that pyroptotic AT2 cells exhibit mitochondrial dysfunction, which drives mtDNA release, ROS-mediated oxidative stress, and subsequent activation of the NLRP3/ASC/caspase-1 pyroptotic pathway. Conversely, SW could upregulate mitochondrial membrane potential, promote ATP production, inhibit mtDNA migration and ROS secretion, block p65 nuclear translocation, down-regulate the expression of mitochondrial iNOS, MT-ND2 and MT-ND4 proteins, and improve mitochondrial function. Inhibition of NLRP3/ASC/Caspase-1 mediated pyroptosis-related signaling pathway, thus improving sepsis-related ALI. Therefore, SW may indirectly regulate the process of pyroptosis by regulating the migration of mtDNA and oxidative damage, and the protective effect is time-dependent. Compared with current research, it is found that inhibiting the release of mtDNA, clearing the free mtDNA accumulated in the lungs, and blocking the mtDNA-mediated pathway are viable therapeutic strategies for sepsis-related ALI (3, 36). SW can enhance the uptake of mitochondria into cells. When exogenous mitochondria are absorbed into cells, they usually fuse with endogenous mitochondria, which may reduce the activation of inflammatory signals downstream of NLRP3 mediated by mtDNA migration and oxidative damage. Moreover, SW-assisted mitochondrial therapy plays a protective role in the morphological structure of lung tissue and prevents ARDS-induced lung injury (18).

SW has certain prospects and advantages in treating and repairing inflammatory injuries associated with respiratory system diseases. This study definitively demonstrates the direct protective effect of SW on the mitochondrial function of AT2 cells and uncovers the intervention mechanism of SW in sepsis-related ALI. The integration of physical therapies, such as SW, with routine basic clinical treatments, can reduce or avert the risks of antibiotic resistance and the immunosuppression associated with anti-inflammatory drugs, including glucocorticoids. A series of energy density parameters were explored through pre-experiments to screen the effective and safe energy density. The parameters of SW are adjustable, and its energy intensity and frequency can be optimized to suit different stages of disease. Additionally, in the future, SW may be used in combination with mitochondrial-targeted drugs, such as MitoQ (an antioxidant), to enhance mitochondrial protection (18).

Nevertheless, this study has certain limitations. Firstly, most current studies on SW do not incorporate positive controls. This study primarily focuses on exploring the intervention effects and underlying mechanisms of SW, but it also lacks a positive control that aligns with the SW treatment protocol. Differently, we introduced a sham control for SW to confirm its safety. In future research, targeted positive controls, target inhibition, or agonists will be added to the research on the intervention target of SW. Secondly, through the comparison of lung tissue structure in control mice at 24h and 48h after SW intervention, mild abnormalities of lung tissue pathology were detected, indicating that SW is safe within the low-energy application range. However, the long-term prognosis still warrants further attention. Such as long-term survival rate, pathological injury repair and functional recovery are urgent clinical problems to be solved, which can also provide more reference and evidence for the clinical transformation and application of SW in the future.

In short, this study also explores a new intervention method for sepsis-induced acute lung injury, which has been initially used in both *in vivo* and *in vitro* studies. The clinical application and transformation of SW in the treatment of lung diseases hold promising prospects, but they also present numerous challenges. At the level of pathological mechanism research, mitochondrial damage is the initiating factor of sepsis, and the related indicators have important clinical diagnostic and predictive value. Clinical studies have found no significant difference in the mtDNA level between patients with sepsis who survived and those who died within 1 day and 3 days. If the levels of mtDNA were reduced by day 7, these patients would survive. But if mtDNA persists and damage persists, the patient’s risk of death increases (37). The release of mtDNA serves as a link between mitochondrial dysfunction and amplified inflammation, while pyroptosis is a key factor leading to lung damage. SW may play a therapeutic role by targeting this chain, providing an important foundation for clinical translation at the level of pathological mechanism. Moreover, it is essential to further clarify the molecular target of SW in alleviating sepsis-related acute lung injury, the mechanism of action on different cell types, and its dynamic regulatory network. At the same time, it is necessary to enhance the safety evidence of SW intervention in more advanced animal models of acute lung injury. For example, SW can protect against acute ischemia-reperfusion injury of the pig heart (38). In clinical practice, it is safe to use low-intensity SW in refractory angina pectoris, erectile dysfunction and other diseases (39, 40). However, the clinical application of SW in patients with acute lung injury due to sepsis requires strict optimization of parameters and further verification of its safety and mechanism. The available safety data may support its trial in non-critical patients, but it needs to be combined with individualized assessment.

In conclusion, this study demonstrated that SW ameliorates sepsis-induced ALI by repairing mitochondrial function, inhibiting mtDNA migration and the activation of the NLRP3 inflammasome, blocking the process of cell pyroptosis, and interrupting the crosstalk between mitochondrial dysfunction and the pyroptosis axis. These findings provide molecular-level mechanistic evidence supporting the clinical translation of SW while expanding its therapeutic potential in modulating immune-inflammatory responses in pulmonary disorders.

## Data Availability

The raw data supporting the conclusions of this article will be made available by the authors, without undue reservation.
