# Supplementary material for: Low-dose extracorporeal shock wave attenuates sepsis-related acute lung injury by targeting mitochondrial dysfunction and pyroptosis crosstalk in type II alveolar epithelial cells
Source: Front Immunol. 2025 Aug 21;16:1637378. doi: 10.3389/fimmu.2025.1637378 (PMC12409442; doi:10.3389/fimmu.2025.1637378)
Supplement: Supplementary file 1 [file Supplementaryfile1.docx]

**Supplementary Material**

1. Original Western Blot image of animal samples


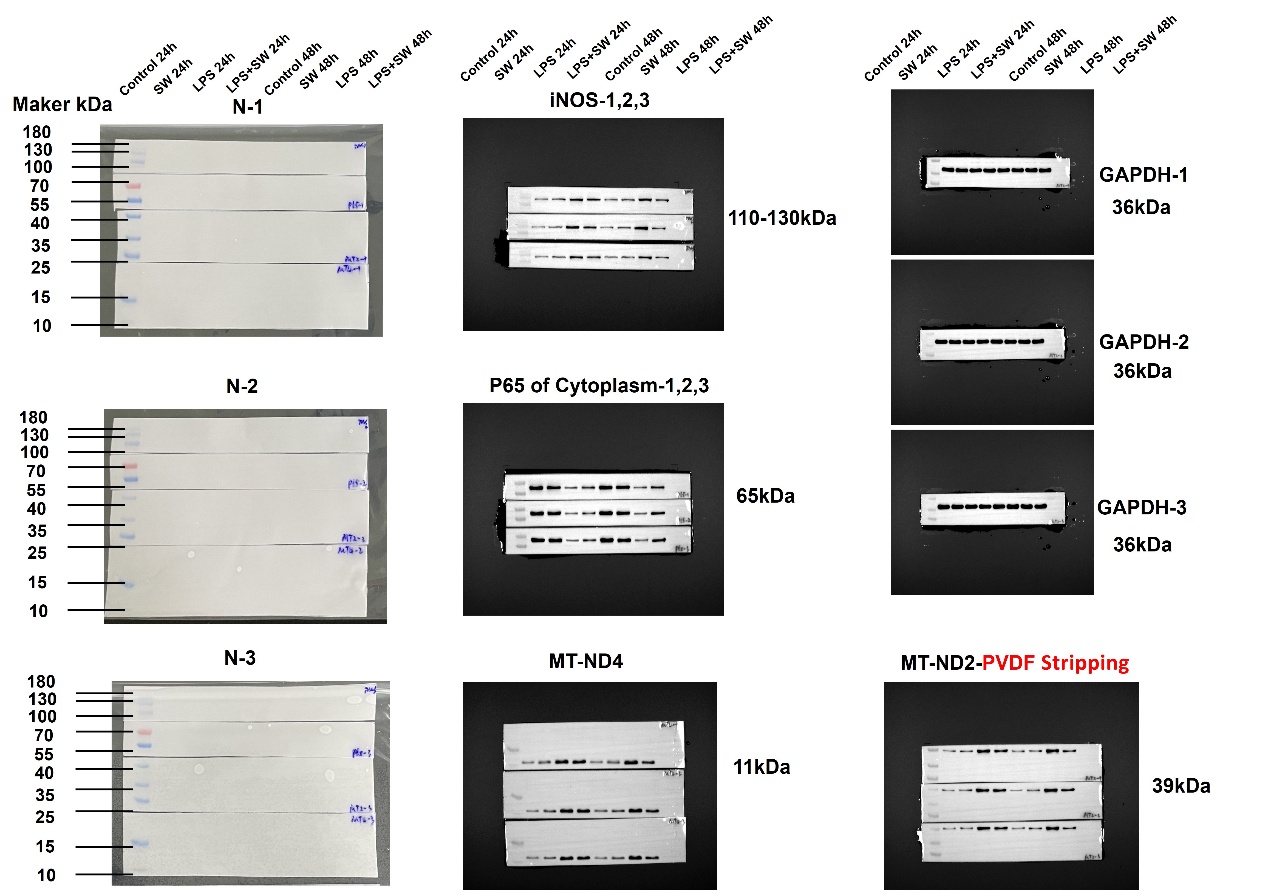


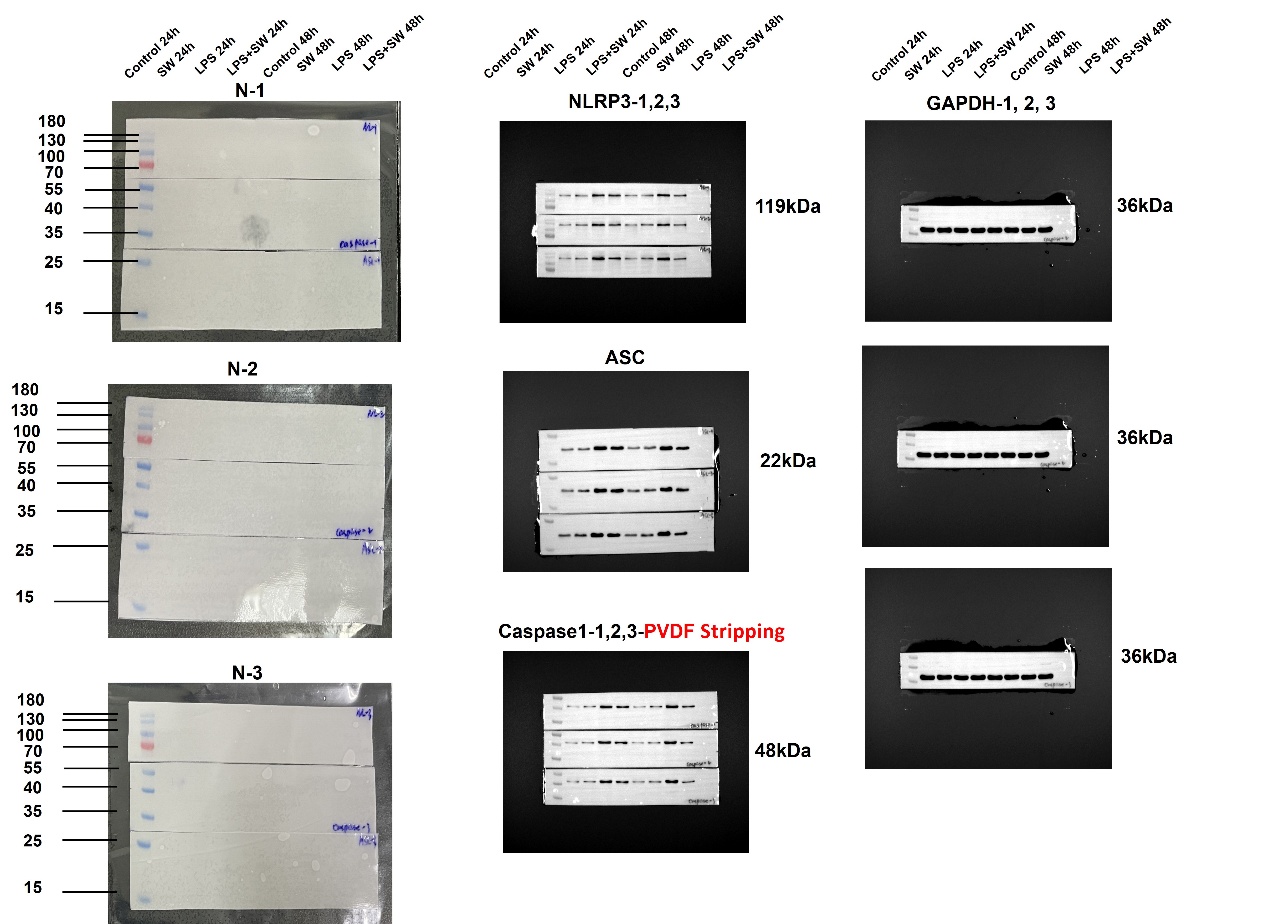


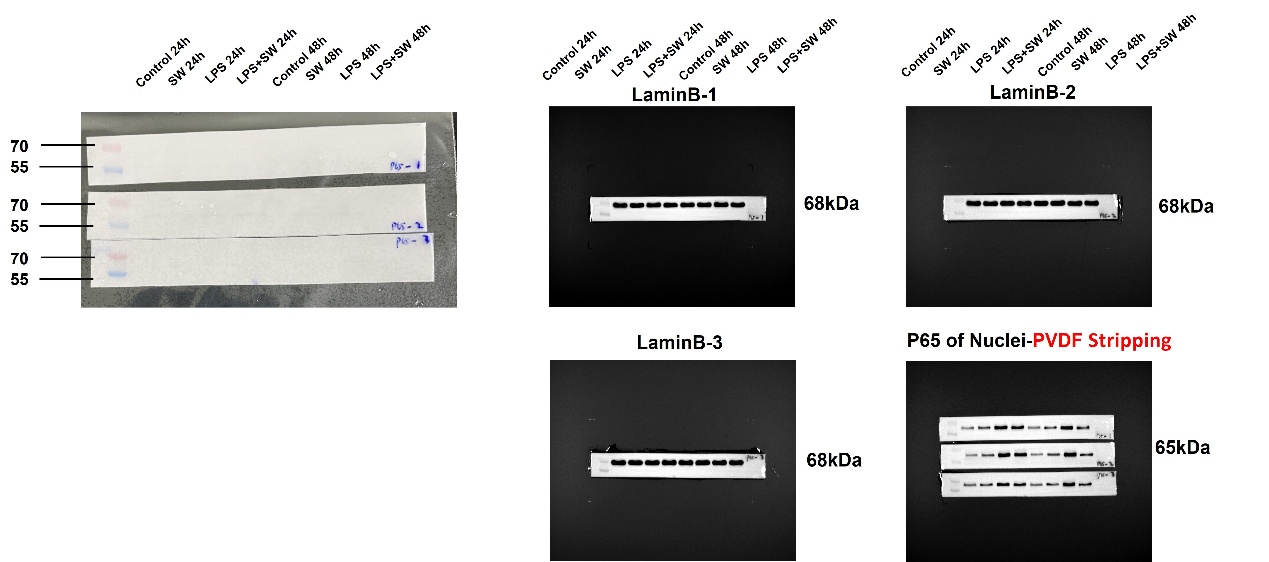


2. Original Western Blot image of AT2 cells samples


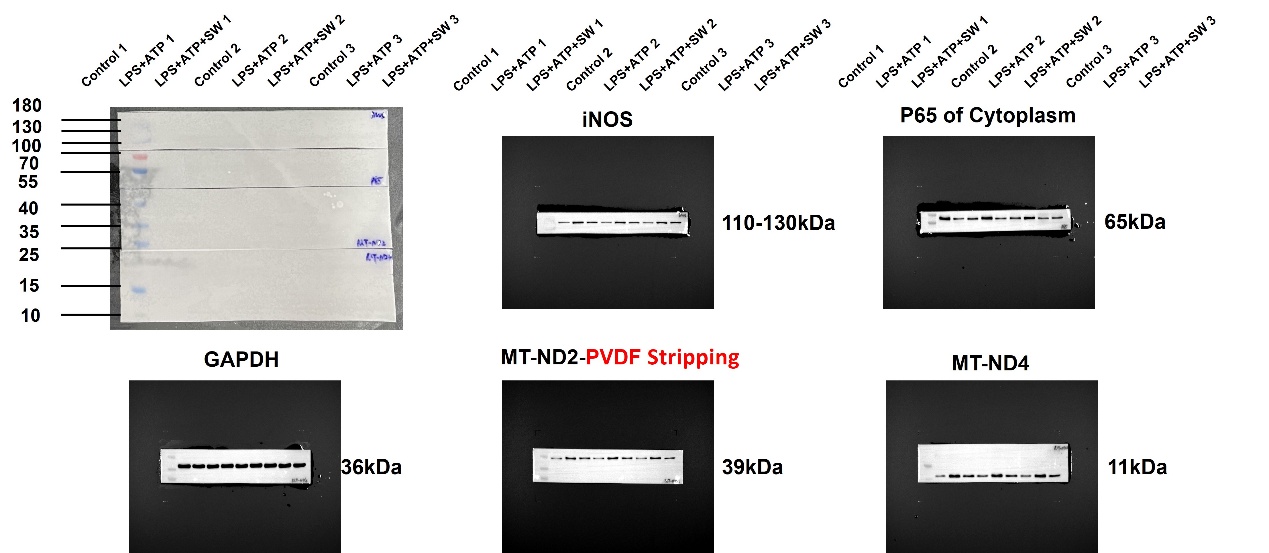


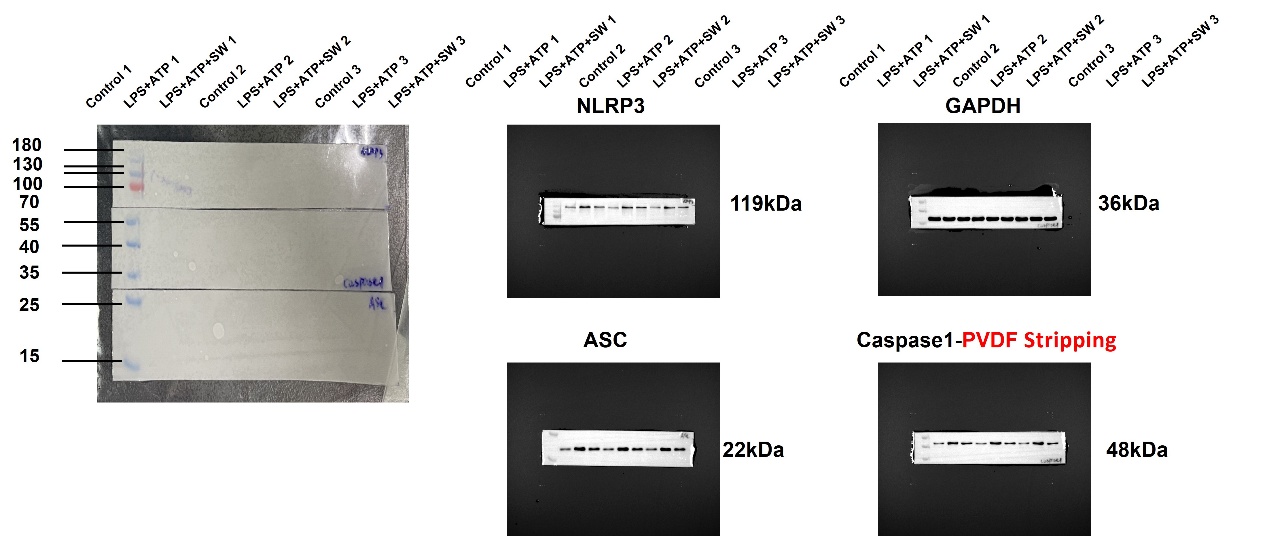


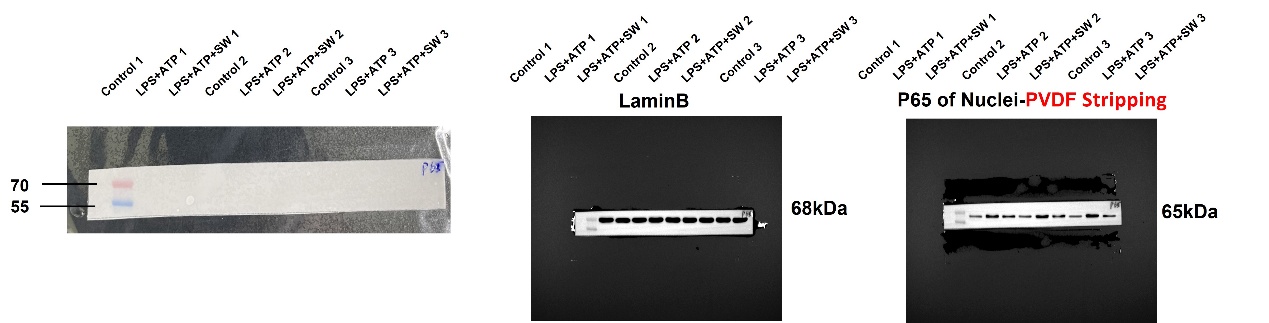


2. CCK8 results of screening parameters for SW intervention


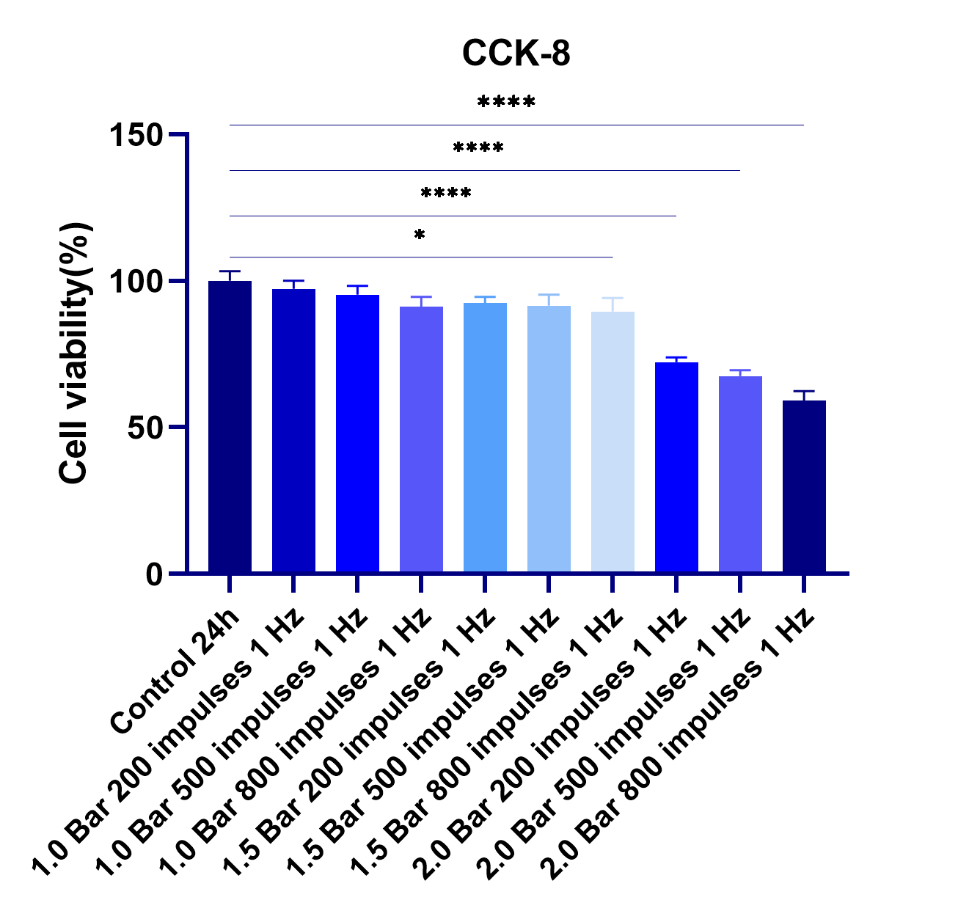


Data are expressed as mean ± SD and analyzed by One-way ANOVA. ns, P > 0.05; * P < 0.05; *** P < 0.001; **** P < 0.0001.

AT2 cells in good condition were collected into 10 sterilized 5 mL polypropylene tubes; one tube served as a blank control, and nine tubes of cells were intervened with SW using different intervention parameters to reduce crossover effects. After intervention, the cells were seeded in 96-well plates (100 μL/well, 4 × 10^3 cells/well) and incubated for 24 h at 37 °C in a 5% CO_2_ incubator. Untreated AT2 cells were used as control, and different intervention parameters of SW were screened: 1.0 Bar ×200 impulses with 1 Hz; 1.0 Bar ×500 impulses with 1 Hz; 1.0 Bar ×800 impulses with 1 Hz; 1.5 Bar ×200 impulses with 1 Hz; 1.5 Bar ×500 impulses with 1 Hz; 1.5 Bar ×800 impulses with 1 Hz; 2.0 Bar ×200 impulses with 1 Hz; 2.0 Bar ×500 impulses with 1 Hz; 2.0 Bar ×800 impulses with 1 Hz. After 24 hours of intervention, 10 μL CCK-8 reagent was added to each well and incubated at 37 °C for 2 hours. The absorbance value was measured at 450 nm using a microplate reader. The maximum intervention parameters screened to avoid affecting AT2 activity were 500 impulses at a frequency of 1 Hz, with an energy intensity of 1.5 Bar.
